# A broad-spectrum SARS-CoV-2 RBD vaccine with selected high-impact mutations and novel adjuvant induces durable T cell response and broad protection in mice

**DOI:** 10.3389/fcimb.2025.1690554

**Published:** 2026-01-30

**Authors:** Xianying Chen, Yuzhen Zhang, Shimin Yang, Yan Chen, Shengnan Qian, Zhen Zhang, Qianyun Liu, Chengbao Ma, Weiyi Yu, Jiangpeng Feng, Jiejie Liu, Ming Guo, Zhixiang Huang, Xin Wang, Jie Chen, Haiyan Zhao, Huan Yan, Ke Lan, Yu Chen, Li Zhou

**Affiliations:** 1 State Key Laboratory of Virology and Biosafety, Animal Bio-safety Level III Laboratory, College of Life Sciences and Frontier Science Center for Immunology and Metabolism, Wuhan University, Wuhan, China; 2 Jiangsu Taipure Biopharmaceuticals Co., Ltd., Taizhou, China

**Keywords:** CpG adjuvant, high-impact mutations, SARS-CoV-2, subunit vaccine, variants of concern

## Abstract

**Introduction:**

The emergence of new SARS-CoV-2 variants with immune evasion capabilities underscores the importance of developing a broad-spectrum and effective vaccine. The receptor binding domain (RBD) of the Spike protein has been widely utilized in vaccine due to its high immunogenicity. However, the Spike protein, particularly the RBD region, exhibits significant variability in the evolution of SARS-CoV-2, leading to viral immune evasion and reduced vaccine effectiveness.

**Methods:**

A broad-spectrum antigen (M5-RBD) was developed via mutation patching, incorporating key high-impact mutation sites (K417T, L452R, T478K, E484K, N501Y). Additionally, extra mutations (N440K or G446S) were introduced into M5-RBD to evaluate their impact on immune response. M5-RBD was further combined with a novel CpG adjuvant HP007 for immunization.

**Results:**

M5-RBD elicited high titers of broad-spectrum neutralizing antibodies against SARS-CoV-2 wild-type and various variants (Delta, Omicron BA.1, BA.2, BA.2.75, BA.5, BF.7, BQ.1.1, XBB, EG.5, JN.1, KP.3 strains). Introduction of N440K or G446S significantly diminished the immune response to viral strains. When combined with HP007 adjuvant, M5-RBD induced efficient and durable T cell responses, providing protection to K18-hACE2 KI mice against lethal infections with both wild-type and Omicron BA.2 strains.

**Discussion:**

Rationally designed with key high-impact mutation sites, M5-RBD effectively overcomes SARS-CoV-2 variant immune evasion and elicits broad-spectrum neutralizing antibodies. The combination with HP007 adjuvant enhances immune protection, providing a promising strategy for the development of next-generation COVID-19 vaccines.

## Introduction

SARS-CoV-2 exhibits high transmissibility and has rapidly spread worldwide, continuously evolving into multiple variants. Currently, SARS-CoV-2 is responsible for more infections and affected regions than SARS and MERS, posing significant threats to global public health and economic development.

Since the onset of the pandemic, five variants of concern (VOCs) for SARS-CoV-2 have emerged, as designated by the World Health Organization: Alpha (B.1.1.7), Beta (B.1.351), Gamma (P.1), Delta (B.1.617.2), and Omicron (B.1.1.529) (Chen et al., 2022). Among these VOCs, the Omicron variant has undergone continuous evolution, resulting in multiple sub-lineages. VOCs exhibit enhanced transmissibility or virulence and can evade the neutralizing effects of antibodies generated through natural infection or vaccination, thereby diminishing the effectiveness of therapeutics and vaccines (Ma et al., 2022). These emerging variants present challenges in managing the disease and underscore the necessity for ongoing surveillance and research to understand their implications for public health measures. Consequently, the development of drugs for the prevention and treatment of SARS-CoV-2 infection has become a primary objective for scientists worldwide in recent years.

Updating existing vaccines to address newly emerging variants has become a common strategy. However, the continuous evolutionary mutations have led to the emergence of several highly transmissible variant strains. Notably, Convergent evolution has been observed in the receptor binding domain (RBD), indicating an increasing concentration and similarity of mutation sites. This phenomenon may be attributed to the “immune imprinting” effect, which reduces the diversity of antibody epitopes generated in response to breakthrough infections. Consequently, there is a tendency for immune pressure to become more centralized. For instance, the Alpha, Beta, Gamma, and Omicron variants exhibit a key mutation, N501Y, in the RBD of the spike protein, which has been reported to increase viral transmissibility by 40% to 70% (Davies et al., 2021). Additionally, the Beta, Gamma, and Omicron variants possess two further RBD mutations: E484 (E484K in Beta and Gamma; E484A in Omicron) and K417 (K417N in Beta and Omicron; K417T in Gamma). The Delta, Omicron BA.4/BA.5, BF.7, and BQ.1 variants also contain L452R mutations. Numerous mutations found in Omicron sub-variants, such as R346T, S371L/F, T376A, D405N, K440T, G446S, N460K, G496S, and others, have been shown to be associated with antibody escape (Nutalai et al., 2022; Kannan et al., 2022; Ao et al., 2023). These mutations may confer immune evasion capabilities against antibodies induced by prototype vaccines and natural infections (Hoffmann et al., 2021; Cele et al., 2021). Therefore, designing mutation-chimeric vaccines based on these high-frequency mutations, which potentially play a significant role in immune evasion, is an essential strategy for the development of broad-spectrum vaccines.

During the SARS-CoV pandemic, it was discovered that only antibodies targeting the spike (S) protein could effectively neutralize the virus and prevent infection (Buchholz et al., 2004). Consequently, many early-stage SARS-CoV-2 vaccine candidates incorporated at least a portion of the S protein, with some specifically targeting the S1 or RBD regions (Jeyanathan et al., 2020). While limited research has suggested the potential of the nucleocapsid (N) protein as a vaccine candidate (Yu et al., 2023), the advantages of the RBD—including its smaller size and efficient expression—over the full-length S protein position it as the most promising vaccine antigen (Krammer, 2020). Furthermore, multiple studies have demonstrated that fusing viral antigens to the immunoglobulin Fc fragment not only facilitates proper folding of the fusion protein and enhances B cell responses, but also significantly improves the immunogenicity of vaccines (Zhang et al., 2025; Sun et al., 2021b). Specifically, the Fc-tagged RBD dimer has been shown to elicit enhanced immunogenicity (Sun et al., 2021a).

Adjuvants are essential components of vaccines that enhance the immune response, ensuring both efficacy and safety by reducing adverse events. Aluminum hydroxide (Al(OH)_3_) helps minimize immune-related pathological reactions, while other adjuvants such as MF59, AS03, CpG 1018, and CoVaccine HT initiate an innate immune response and facilitate the overall immune response. Recombinant proteins lack pathogen-associated molecular patterns (PAMPs), which are essential immunostimulatory molecules. Incorporating an adjuvant into a recombinant protein vaccine can effectively activate the innate immune system and trigger local inflammatory responses. This process facilitates the recruitment of immune cells to the injection site, thereby enhancing antigen-specific immunity. Although aluminum is a commonly used adjuvant, its capacity to activate robust immune responses is limited. Research indicates that the co-administration of aluminum adjuvant with CpG adjuvant can significantly improve vaccine immunogenicity. The CpG adjuvants function as a TLR9 agonist, promoting robust activation of B cells and NK cells. Currently, CpG 1018 remains the only approved CpG adjuvant for human use. Therefore, the inclusion of potent adjuvants is essential for improving both the immunogenicity and durability of vaccine-induced protection.

Given these points, to address the need for a vaccine with higher yields, improved immunogenicity, and enhanced efficacy against highly mutated viruses, a broad-spectrum antigen based on the RBD-Fc dimer containing the K417T, L452R, T478K, E484K, and N501Y mutation sites, named M5-RBD, was developed. When combined with the CpG adjuvant HP007, M5-RBD induces durable and stronger humoral and cellular immune responses in mice, efficiently protecting K18-hACE2 mice from lethal doses of both the SARS-CoV-2 wild type and the Omicron BA.2 variant challenge.

## Results

### RBD with selected high-impact mutations was designed as broad-spectrum antigen

**Figure 1:**
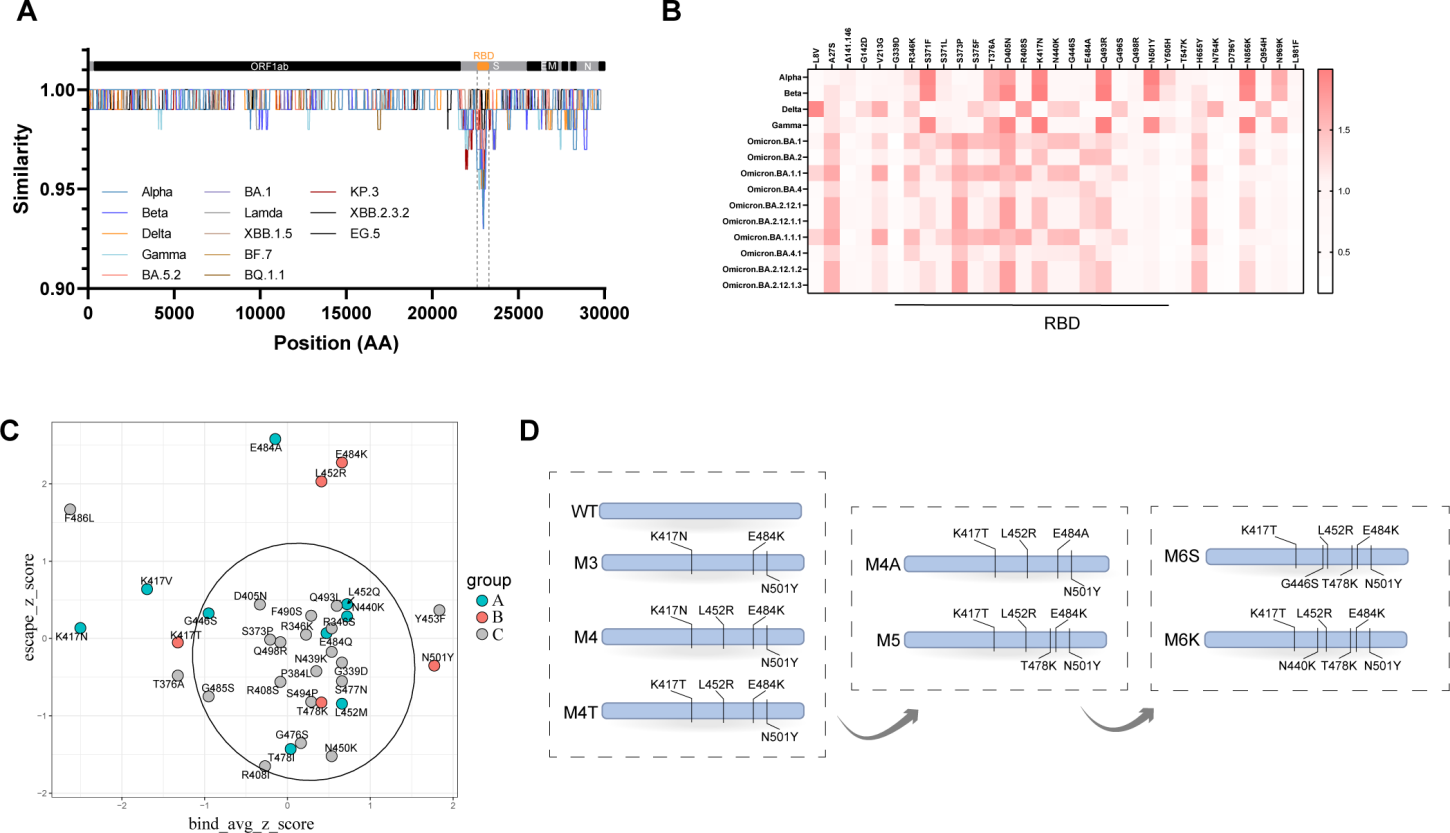
Screening of high-impact mutant RBD antigen. **(A)** SimPlot analysis of nucleotide sequence similarity between SARS-CoV-2 Omicron BA.2 and other variant strains. The upper portion indicates the proteins encoded by SARS-CoV-2, with The orange box highlighting the RBD region. **(B)** Analysis of the Correlation between Antibody Immune Escape and S Protein Mutation Sites. **(C)** Normalization of data on RBD binding to ACE2(X-axis) and the neutralization of antibody escape(Y-axis) using Z-scores.The black circle delineates the region of mutation sites classified as low-impact. **(D)** Process flow for RBD vaccine mutation sites.

Previous studies have indicated that individuals infected with the prototype strain exhibit some degree of immune evasion against mutant strains, including Delta. Notably, the K417, L452, and E484 mutations in the RBD region are closely associated with immune evasion (Liu et al., 2022a). Furthermore, research has demonstrated that the RBD region is particularly crucial for the neutralizing antibody response, with over 90% of antibodies targeting this region (Piccoli et al., 2020). A nucleotide similarity comparison of viral genomes using SimPlot revealed that the RBD region exhibits the greatest variation among different SARS-CoV-2 mutant strains across the entire genome (**Figure 1A**, **Supplementary Figure S1**). We used R programming to analyze the neutralization effects of 10 antibodies across three categories on variant strains and examined the impact of single-point mutations in pseudo-viruses on the neutralizing ability of these antibodies. The results indicated that mutations responsible for the reduced neutralization capacity of antibodies against the variant strains were primarily concentrated in the RBD region,while the regions outside the RBD had a weaker effect on the immune escape ability(**Figure 1B**). Subsequently, we collected published data on the effects of all possible amino acid mutations (3804 mutations) in the RBD on ACE2 binding, as well as data on RBD mutations that lead to immune escape from neutralizing antibodies (434 data points) (Wang et al., 2022, 2021; Starr et al., 2020; Cox et al., 2023). Both datasets were standardized using Z-scores and then subjected to correlation analysis. As shown in **Figure 1C**, the X-axis represents the mutation-induced change in the RBD-ACE2 binding score, and the Y-axis represents the mutation-induced change in the escape score for neutralizing antibodies. We found that most mutation sites (within the black circle, called low-impact mutations) clustered near the origin of both axes, indicating that the majority of mutations in the RBD region had minimal effects on both binding and immune escape. Only a few sites (outside the black circle, called high-impact mutations), such as 417 (including K417N, K417V, K417T), L452R, 484 (including E484K, E484A), and the widely reported N501Y, produced significant effects. Based on these findings, we hypothesize that the vaccine design incorporating both highly variable and conserved sites could potentially yield better efficacy, and we selected several sites (marked in the figure) for further experimental investigation. Accordingly, antigens featuring various combinations of mutation sites, including M3, M4, and M4T antigens, were designed and expressed in 293F suspension cell lines (**Figure 1D**). The Cell supernatants were purified using affinity chromatography and size exclusion chromatography (**Supplementary Figures S2A, B**). The affinity between the recombinant mutant protein RBD-Fc and the hACE2 receptor was assessed employing biolayer interferometry (BLI) technology (**Supplementary Figure S2C**).

### M5-RBD induced broad humoral immunity against SARS-CoV-2 variants

**Figure 2:**
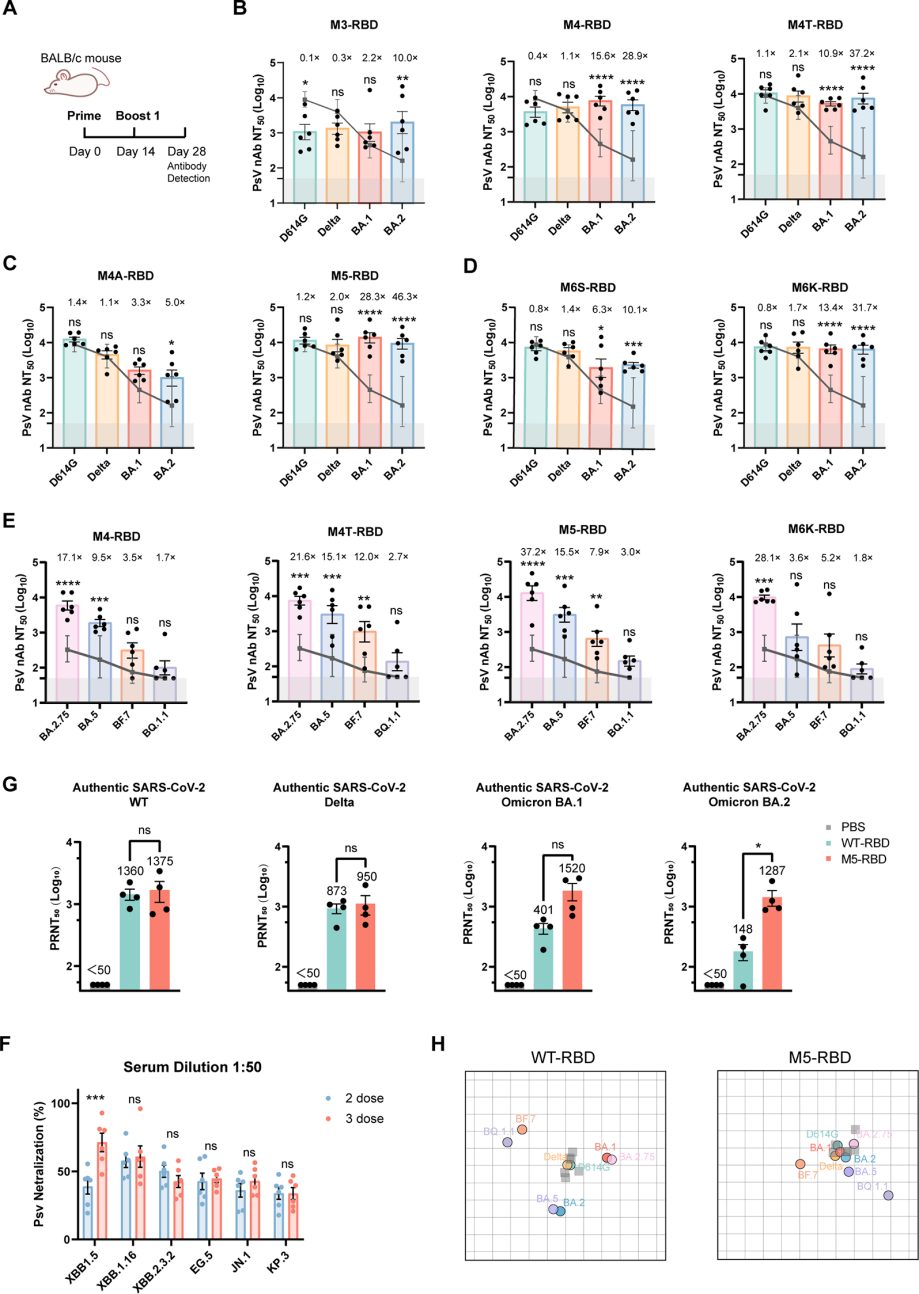
The M5-RBD antigen elicits a broad-spectrum humoral response against mutant strains. **(A)**. Timeline of the mouse experiment. **(B–D)**. nAb Titers of mouse sera (n = 6) immunized with mutant vaccines against D614G, Delta, Omicron BA.1, and BA.2 strains. The dark gray line represents the geometric mean titer (GMT) of nAb induced by the prototype vaccine WT-RBD against the corresponding variant strain. The gray shading indicates the limit of detection (ID_50_ = 50). Data are presented as Mean ± SEM. The fold-change in GMT for each mutant antigen is shown relative to the prototype vaccine. “ns” indicates P > 0.05, “*” indicates P < 0.05, “**” indicates P < 0.01, and “***” indicates P < 0.001 and “****” indicates P < 0.0001. **(E)**. nAb Titers of mouse sera (n = 6) immunized with mutant vaccines targeting the BA.2.75, BA.5, BF.7, and BQ.1.1 strains. **(F)** Heat map representation of the neutralizing activity of one representative mouse serum sample from each group against pseudoviruses. Relative neutralization was determined using a 1:100 antibody dilution. Neutralizing activity of the WT-RBD immunized group was set to 1. Blue color represents increased neutralizing activity of serum samples. **(G)** Serum neutralization assay of authentic SARS-CoV-2 virus using sera from immunized mice. Serum samples were collected from 6–8 week-old BALB/c mice following two immunizations. The mouse serum was serially diluted and incubated with 100 TCID_50_ of authentic SARS-CoV-2 virus for 30 mins, followed by infection of Vero E6 cells. The cells were cultured in methylcellulose medium for an additional 2 hours. After 3 days, the cells were fixed, stained with crystal violet, and counted. The dashed lines indicate 0% and 50% neutralization activity. Data are presented as Mean ± SEM. **(H)** Antigenic maps of WT-RBD and M5-RBD immunized mouse sera. Antigenic maps were generated from serum samples of BALB/c mice immunized with 5 μg of either WT-RBD or M5-RBD protein in two doses. The green, yellow, red, blue, pink, light purple, orange, and gray-purple circles correspond to the D614G, Delta, BA.1, BA.2, BA.2.75, BA.5, BF.7, and BQ.1.1 strains, respectively. strain names are indicated in the same color as their corresponding circles. A translucent gray square represents one serum sample. The spatial antigenic distance (measured in Antigenic Units, AU) between a serum point and a variant circle is a quantitative, non-directional metric derived from neutralization titers. A shorter distance indicates stronger serum neutralizing potency against that variant. Clustering of serum points (implied by proximity) indicates groups with similar antigenic recognition profiles.

BALB/c mice were intramuscularly immunized with 5 μg of RBD mutant protein, adjuvanted with CpG and Al(OH)_3_. Mouse serum was collected two weeks after the second dose to detect the levels of neutralizing antibodies (nAb) against the WT-D614G, Delta, and Omicron BA.1 and BA.2 variants (**Figure 2A**). The results indicated that, compared to WT-RBD, the M3-RBD antigen, which carries the K417N, E484K, and N501Y mutations, elicited relatively lower levels of neutralizing antibodies against Delta (0.3 ×), BA.1 (2.2 ×), BA.2 (10.0 ×), and the prototype strain (0.1 ×). The introduction of the L452R mutation (M4-RBD) resulted in higher levels of neutralizing antibodies against BA.1 (15.6 ×) and BA.2 (28.9 ×) compared to the WT-RBD antigen. Interestingly, while BA.1 and BA.2 do not possess the L452R mutation, the newly identified strains BA.4 and BA.5 reintroduce this mutation. The M4T-RBD antigen, which carries the K417T mutation, demonstrated significant inhibition of Omicron BA.1 (10.9 ×) and BA.2 (37.2 ×), while maintaining its inhibitory effect on the WT-D614G strain (1.1 ×) and the Delta strain (2.1 ×). Thus, M4T exhibited relatively good broad-spectrum immunogenicity (**Figure 2B**).

Based on the M4T-RBD antigen, we introduced two additional significant mutations, E484A and T478K. despite the fact that the majority of mutations at position 484 in the Omicron strains are A rather than K, the neutralizing antibodies (nAb) induced by the M4A-RBD antigen against BA.1 (3.3 ×) and BA.2 (5.0 ×) were significantly reduced (**Figure 2C**). However, upon incorporating the T478K mutation, the M5-RBD antigen exhibited enhanced neutralizing activity against BA.1 (28.3 ×) and BA.2 (46.3 ×). Consequently, the M5-RBD antigen, which carries the K417T, L452R, T478K, E484K, and N501Y mutations, demonstrated the broadest neutralizing activity.

Studies indicate that the N440K and G446S mutations in Omicron partially reduce the neutralizing activity of certain antibodies against Omicron strains (Cao et al., 2022). Therefore, these two mutations were introduced into the M5-RBD, resulting in antigens designated as M6S and M6K, respectively. The M6S-RBD antigen elicited lower levels of antibodies against BA.1 (6.3 ×) and BA.2 (10.1 ×) compared to the M5-RBD. Conversely, the introduction of the N440K mutation into the M5-based antigen (M6K) resulted in higher neutralizing activity against BA.1 (13.4 ×) and BA.2 (31.7 ×), although it was still not as effective as the M5-RBD antigen (**Figure 2D**).

As the omicron sub-lineages variants, such as BA.2.75, BA.5, BF.7, and BQ.1.1, emerged frequently and rapidly, the nAb levels induced by the superior M4-RBD, M4T-RBD, M5-RBD, and M6K-RBD antigens were assessed (**Figure 2E**). Among these, M5-RBD elicited antibody levels against the newly six variants higher than those induced by the WT-RBD antigen (**Figure 2F**), further validating the prospective value of the M5-RBD antigen.

For additional assessment, four mice from both the WT-RBD and M5-RBD immunization groups were randomly selected to perform a live SARS-CoV-2 virus neutralization assay (**Figure 2G**). The results indicated that M5-RBD induced robust antibody neutralization responses in mice against BA.1 and BA.2. Following two immunizations with a 5 μg dose, the plaque reduction neutralization test 50% (PRNT_50_) values were recorded at 1520 and 1287, respectively, which were approximately 3.8 and 8.7 times higher than those induced by the WT-RBD group. Additionally, the M5-RBD group exhibited slightly elevated levels of neutralizing antibodies against the WT and Delta strains compared to the WT-RBD group, with increases of approximately 1.0-fold and 1.1-fold, respectively. Overall, under the conditions of two doses of 5 μg immunization, M5-RBD was able to induce high levels of neutralizing antibodies against the mutated strains, and the results of the authentic virus neutralization assay were consistent with those of the pseudovirus neutralization assay.

Antigenic maps were employed to quantify and visualize neutralization data. The antigenic maps for the WT-RBD and M5-RBD groups were generated to investigate how the sera differentiate between variants. In the WT-RBD Immunization group, serum clustered around the D614G and Delta strains, distinctly separating from other variants (**Figure 2H**). In contrast, serum from the M5-RBD immunization group clustered around D614G, Delta, BA.1, BA.2, BA.2.75, and BA.5. Notably, the antigenic distance between BA.1 and D614G in the M5-RBD group was approximately 5.2 times closer than that observed in the WT-RBD group. Similarly, the antigenic distance between BA.2 and D614G in the M5-RBD group was approximately 3.3 times closer than in the WT-RBD group. These results indicate that M5-RBD induces a broader range of neutralizing antibodies against SARS-CoV-2 variants. Overall, M5-RBD eliciting high levels of neutralizing antibodies against mutant strains.

### HP007 elicited broad and durable humoral and cellular immunity in mice

**Figure 3:**
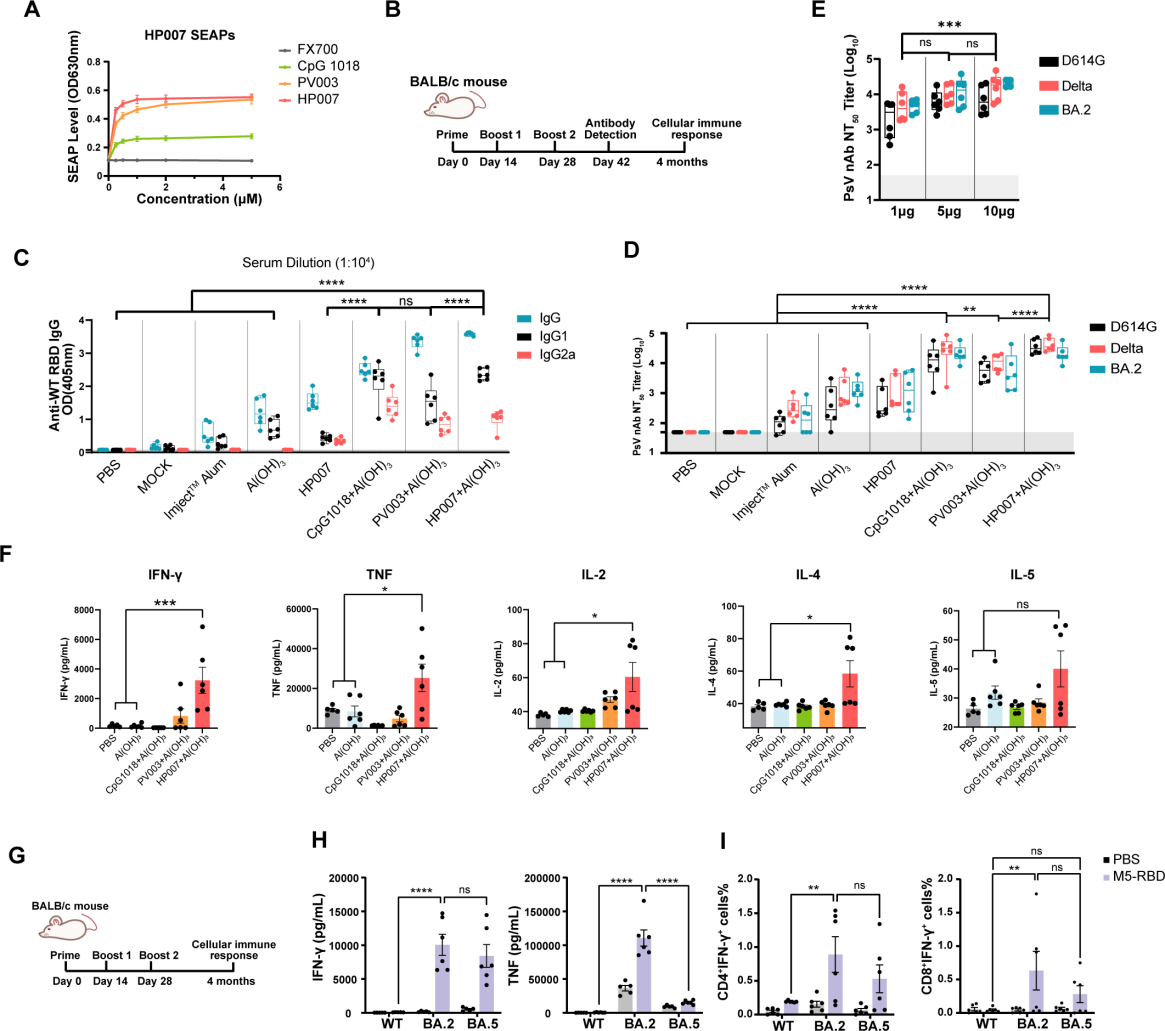
HP007 plus Al(OH)_3_ elicits broad and durable humoral and cellular response. **(A)** The CpG adjuvant activates the TLR9 pathway. Various concentrations of the adjuvant were added to HEK-Blue™ mTLR9 cells. After 24 hours, the cell supernatant was collected and mixed with QUANTI-Blue™ detection solution, followed by incubation at 37 °C for a duration ranging from 10 minutes to 6 hours. SEAP levels were measured using an enzyme-linked immunosorbent assay (ELISA) at a wavelength of 630 nm. **(B)** Timeline of the mouse experiment. BALB/c mice aged 6–8 weeks were immunized three times, and Blood samples were collected two weeks after the final immunization. After three immunizations, mouse spleen cells were collected. **(C, D, F)** Formulation of immunization groups: All groups received the M5-RBD protein as the common antigen. Groups are as follows: PBS, MOCK (M5-RBD protein alone, without adjuvant), and M5-RBD protein formulated with each of the following adjuvants, only the adjuvant information is labeled in the figure. **(C)** The levels of mouse IgG, IgG1, and IgG2a antibodies induced by the adjuvant were measured using ELISA. The gray shaded area indicates the lower limit of antibody detection. **(D)** Adjuvant-induced levels of neutralizing antibodies in mice were assessed. the levels of neutralizing antibodies against pseudoviruses expressing D614G, Delta, and Omicron BA.2 were measured using a pseudovirus assay system. The gray shaded area represents the lower limit of neutralizing antibody detection (ID_50_ = 50). **(E)** Neutralizing antibody levels activated by different M5-RBD antigen doses with HP007 plus Al(OH)_3_. BALB/c mice aged 6–8 weeks were immunized twice, and blood samples were collected two weeks after the second immunization. the levels of neutralizing antibodies against pseudoviruses expressing D614G, Delta, and Omicron BA.2 were measured using a pseudovirus assay system. **(F)** The HP007 adjuvant effectively activates cellular immunity. After three immunizations, mouse spleen cells were isolated and stimulated with His-tagged Omicron BA.2 protein. After 48 hours, the cell supernatant was collected, and cytokine levels were measured using the CBA assay kit. “ns” indicates P > 0.05, “**” indicates P < 0.01, and “****” indicates P < 0.0001. **(G)** Timeline of the mouse experiment. 6–8 week-old BALB/c mice (n=6) were immunized three times with 10 μg of M5-RBD adjuvanted with HP007 with Al(OH)_3_, with a two-week interval between immunizations. Mouse spleen cells were collected four months after the initial immunization. **(H, I)**. The spleen cells were stimulated with WT RBD, Omicron BA.2 RBD, or Omicron BA.5 RBD. IFN-γ and TNF expression in M5-RBD immunized mouse spleen cells stimulated with WT and Omicron RBD proteins were detected using the CBA assay kit after 48 hours. **(I)** The presence of IFN-γ^+^CD4^+^ and IFN-γ^+^CD8^+^ cells in M5-RBD-immunized mice was assessed following stimulation with either WT or Omicron RBD proteins. The Data are presented as Mean ± SEM. The notation ‘ns’ denotes P > 0.05, ‘**’ indicates P < 0.01, and ‘****’ indicates P < 0.0001.

In our study, we compared two novel CpG adjuvants, PV003 and HP007, with CpG 1018. The results demonstrated that HP007 exhibited dose-dependent activation of mTLR9, with peak activation occurring at a concentration of 1 μM, followed by a plateau phase. In contrast, CpG 1018 and PV003 displayed relatively lower levels of activation. Furthermore, the reverse sequence FX700 did not show significant activation of mTLR9 (**Figure 3A**).

To compare the IgG and nAb titers elicited by CpG adjuvanted vaccines, BALB/c mice were immunized with the M5-RBD protein, which was adjuvanted with various formulations (**Figure 3B**). The results indicated that there was no significant increase in IgG levels for the immunized antigen group without adjuvant (M5-RBD) compared to the PBS immunization control group (**Figure 3C**). Among the tested groups, the combination of HP007 and Al(OH)_3_ yielded the highest IgG levels. When the serum was diluted to 1:10^4^, the optical density (OD) values were 1.1 times greater than those of the CpG1018 plus Al(OH)_3_ group, and approximately 3.0 times and 3.4 times higher than those of the HP007 group and Al(OH)_3_ group, respectively. Furthermore, there were minimal IgG2a antibodies detected in the Al(OH)_3_ adjuvant group, whereas all three CpG adjuvants in the combination groups were able to activate a certain level of IgG2a, suggesting that CpG adjuvants have the potential to stimulate a Th1 -type cellular immune response.

Furthermore, the neutralizing antibody titers were assessed (**Figure 3D**). The results indicated that the NT_50_ values for the group without adjuvants were all below 50, which was similar to the PBS-immunized group. The combination of HP007 or PV003 with Al(OH)_3_ adjuvant significantly enhanced immunogenicity compared to Al(OH)_3_ alone, with geometric mean titers (GMT) for the D614G pseudovirus increased by 99-fold and 13-fold, respectively, while the CpG 1018 combination group demonstrated a 28-fold increase. The Imject™ Alum group exhibited relatively lower activity, with a GMT of only 97. When used alone, M5-RBD adjuvanted with HP007 achieved a comparable GMT to the Al(OH)_3_ group, with only a 1.3-fold increase. Moreover, the neutralization activity of these groups against Delta and BA.2 pseudoviruses displayed consistent trends with the neutralization observed against the D614G pseudovirus. Notably, The Imject™ Alum group exhibited slightly higher levels of neutralizing antibodies compared to the PBS and M5-RBD groups. In comparison to the Al(OH)_3_ group, the CpG1018, PV003, and HP007 plus Al(OH)_3_ groups enhanced the titers by 23-fold, 10-fold, and 40-fold, respectively. Additionally, even at a low dose of 1 μg antigen, the nAb levels in the HP007 adjuvanted plus Al(OH)_3_ group were higher than those in the Al(OH)_3_ adjuvanted group (**Figure 3E**). In conclusion, the novel CpG adjuvant, HP007, significantly enhances immunogenicity more than the approved CpG 1018 adjuvant.

The CpG plus Al(OH)_3_ groups activated IgG1 and IgG2a, suggesting that CpG adjuvants have the potential to enhance cellular immunity. To further compare the effects of these three CpG -adjuvanted groups on the activation of cellular immunity, lymphocytes were isolated from the spleen and subsequently analyzed using intracellular cytokine staining (ICS) and Cytometric bead array (CBA). The results (**Figure 3F**) indicated that, similar to the Al(OH)_3_ adjuvant group, low levels of IgG2a antibodies were produced, and the levels of IFN-γ and TNF were also relatively low, with no significant differences observed when compared to the PBS group. However, IL-5 was induced, suggesting that the Al(OH)_3_ adjuvant elicited a Th2-type cellular immune response. Unexpectedly, the CpG 1018 group did not exhibit significant cytokine expression. This may be attributed to a delay in the timing of cytokine detection, which could have resulted in undetected levels of cellular immunity. In comparison, while PV003 induced relatively low levels of cytokine expression, HP007 combined with Al(OH)_3_ activated significantly higher levels of cytokine expression.

Moreover, we further investigated the efficiency of M5-RBD adjuvanted with HP007 plus Al(OH)_3_ in activating T cell responses against variants. BALB/c mice were immunized with 10 μg vaccine three times, and lymphocytes were isolated from the spleen two months after the final immunization (**Figure 3G**). The results indicated that IFN-γ production following stimulation with Omicron BA.2 protein was 125.5-fold and 105.1-fold higher compared to stimulation with WT protein and Omicron BA.5 protein, respectively (**Figure 3H**). Additionally, TNF levels after stimulation with Omicron BA.2 protein were 218.1-fold and 30.4-fold higher than those following stimulation with WT protein and Omicron BA.5 protein, respectively. Although the cellular immune response induced by WT protein stimulation was not as robust as that induced by Omicron proteins, WT protein stimulation did increase the levels of IFN-γ and TNF in the M5-RBD immunization group relative to the PBS immunization group by 2.7-fold and 3.2-fold, respectively. WT-RBD, Omicron BA.2-RBD, and Omicron BA.5-RBD proteins were capable of activating IFN-γ^+^ CD4^+^ cells (**Figure 3I**). The number of activated cells increased by 4.1-fold, 5.8-fold, and 8.1-fold compared to the PBS group, respectively. Similarly, immunized mice produced a significant number of IFN-γ^+^ CD8^+^ cells targeting Omicron BA.2 RBD and BA.5 RBD proteins, with the strongest cellular immune response observed against the Omicron BA.2 RBD protein. These results indicate that M5-RBD can effectively activate cellular immune responses against mutant strains in mice.

### M5-RBD adjuvanted with HP007 plus Al(OH)_3_ provided complete protection against SARS-CoV-2 WT and Omicron challenge

**Figure 4:**
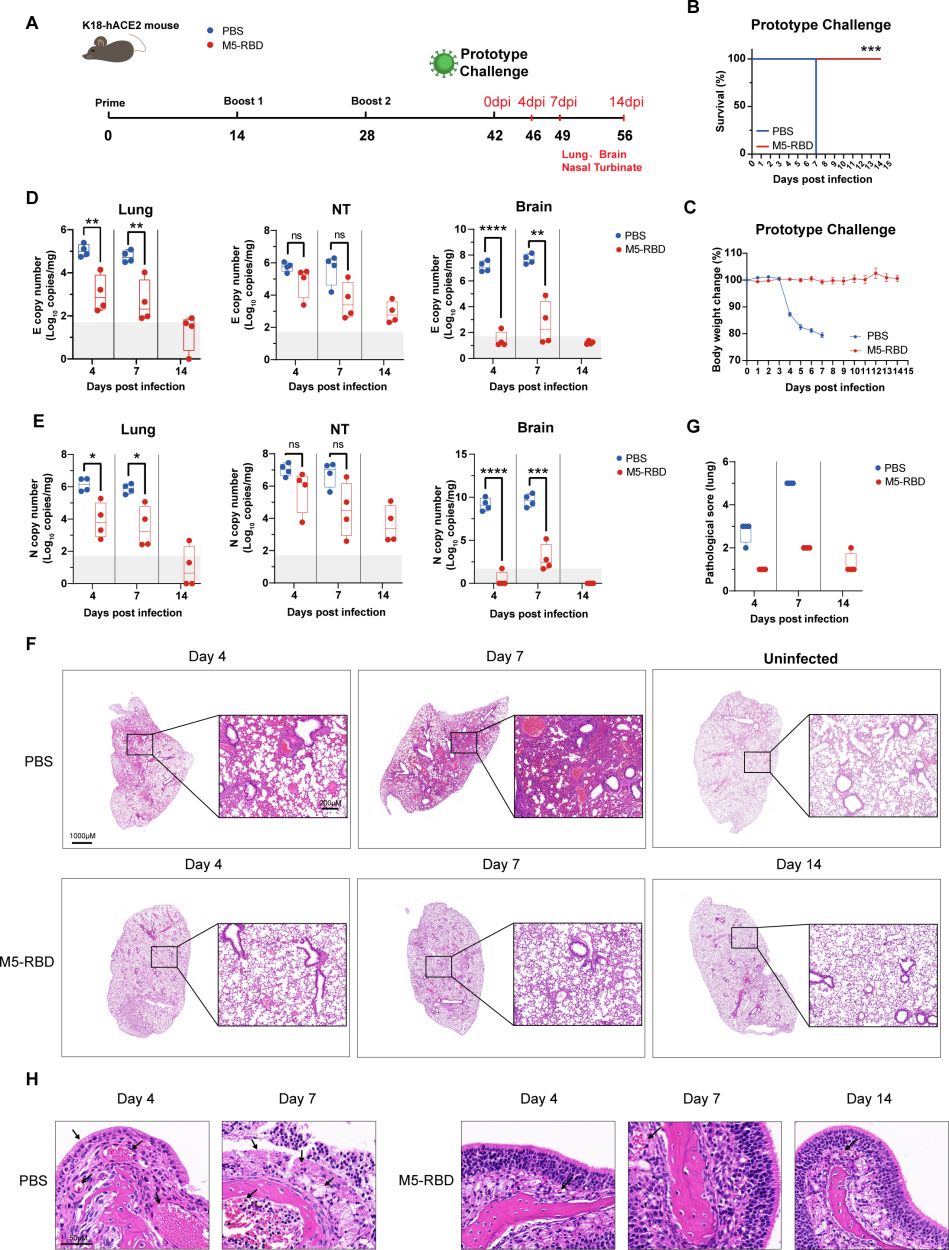
M5-RBD adjuvanted with HP007 plus Al(OH)_3_ provides effective protection for K18-hACE2 mice against SARS-CoV-2 WT infection. K18-hACE2 mice, aged 6–8 weeks, were immunized three times before being subjected to intranasal infection with 250 PFU of the SARS-CoV-2 WT strain. **(A)** Timeline of mouse experiment. **(B)** Survival rate of mice following the viral challenge. **(C)** Weight changes in mice post-challenge, with the percentage change in body weight relative to 0 dpi represented as Mean ± SEM. **(D)** Copy numbers of the SARS-CoV-2 E gene in the lung, nasal turbinate, and brain tissues of the mice after the viral challenge. **(E)** Copy numbers of the SARS-CoV-2 N gene in mouse lung, nasal turbinate, and brain tissues following the challenge. **(F)** At the corresponding time points, mice were euthanized (or had succumbed to the infection), and the left lung was collected. After fixation in 4% paraformaldehyde for three days, the samples were prepared for paraffin section, followed by H&E staining. The left panel provides an overall view of the lung, while the right panel displays a 5-fold magnification. Scale bars are indicated as 1000 μm and 200 μm, respectively. **(G)** Mouse lung sections stained with H&E to assess lung pathology. **(H)** Histopathological sections of the nasal turbinate in K18-hACE2 mice that were immunized with the SARS-CoV-2 WT challenge vaccine. The PBS group (n = 8), while the vaccine-immunized group (n = 12). Asterisks indicate statistical significance, with “***” representing P < 0.001.

**Figure 5:**
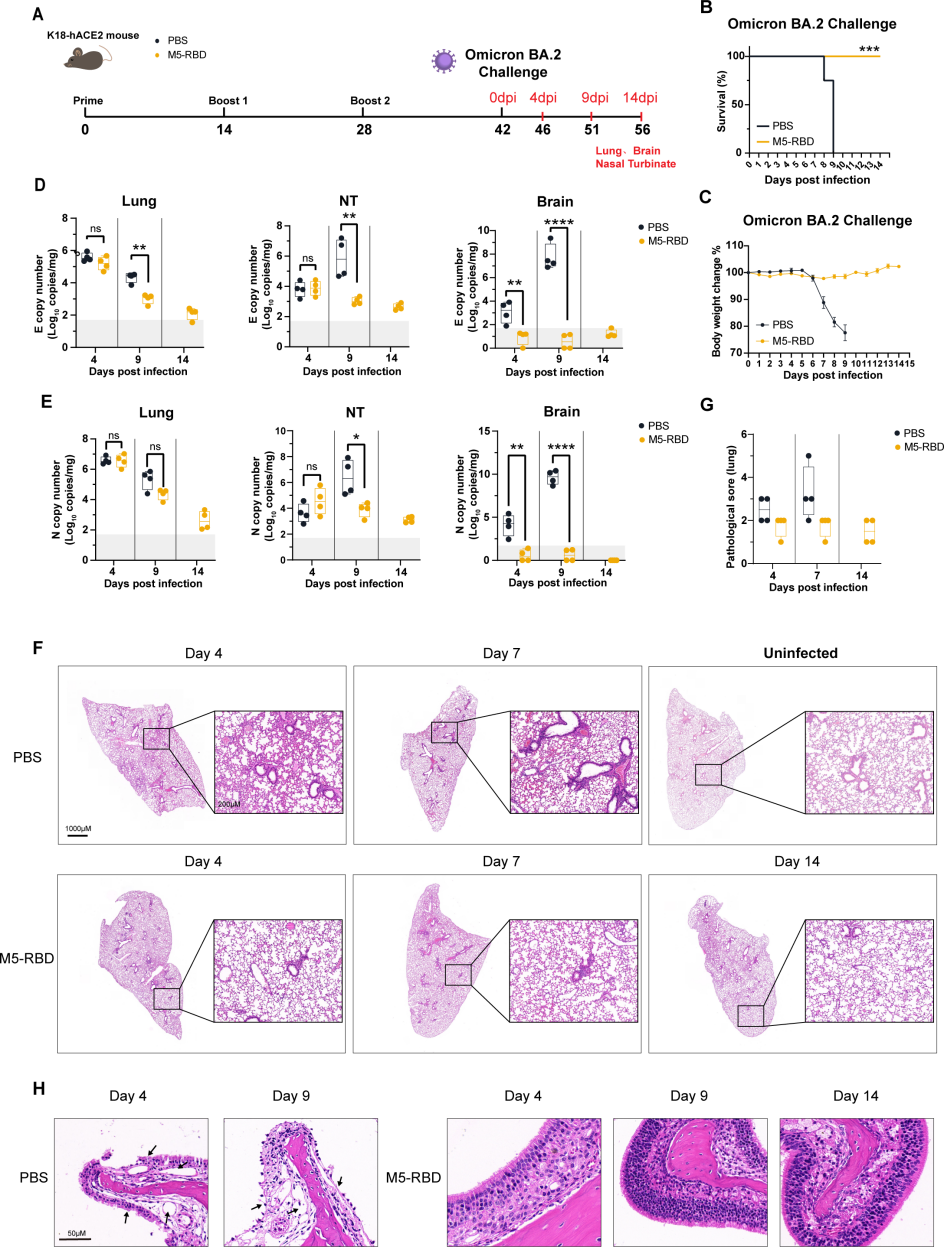
M5-RBD adjuvanted with HP007 plus Al(OH)_3_ effectively protects K18-hACE2 mice against SARS-CoV-2 Omicron BA.2 infection. K18-hACE2 mice, aged 6–8 weeks, were immunized three times before being subjected to intranasal infection with 5000 PFU of the SARS-CoV-2 Omicron BA.2 strain. **(A)** Timeline of the mouse experiment. **(B)** Survival rate of mice following the viral challenge. **(C)** Weight changes in mice post-challenge, with the percentage change in weight relative to 0 dpi expressed as Mean ± SEM. **(D)** Copy numbers of the SARS-CoV-2 E gene in the lung, nasal turbinate, and brain tissues of the mice after the viral challenge. **(E)** Copy numbers of the SARS-CoV-2 N gene in these same tissues. **(F)** At the corresponding time points, mice were either euthanized or had succumbed to the infection, and the left lung was collected. Following fixation in 4% paraformaldehyde for 3 days, the samples were prepared for paraffin section, followed by H&E staining. The left panel displays an overall view of the lung, while the right panel shows a 5-fold magnification, with Scale bars indicating 1000 μm and 200 μm, respectively. **(G)** the evaluation of lung pathology through scoring of H&E stained lung sections. **(H)** Histopathological sections of the nasal turbinate in K18-hACE2 mice immunized with the SARS-CoV-2 WT challenge vaccine. The PBS group (n = 8), vaccine-immunized group (n = 12) Asterisks indicate statistical significance, with ‘***’ representing P < 0.001.

To evaluate the vaccine’s efficacy in protecting mice against SARS-CoV-2 infection, 6-to 8-week-old K18-hACE2 mice were immunized with three doses of 5 μg M5-RBD adjuvanted with HP007 plus Al(OH)_3_ via intramuscular injection, with a two-week interval between doses. immunizations. PBS was administered as a placebo control. Following immunization, the mice were intranasally infected with 250 PFU of SARS-CoV-2 WT (**Figure 4A**) or 5000 PFU of SARS-CoV-2 Omicron BA.2 (**Figure 5A**) (Zhang et al., 2024).

Daily observations were conducted to monitor body weight and survival rates post-infection. In the control group, infection with the SARS-CoV-2 WT strain progressed rapidly, resulting in a sharp decline in body weight observed at 4 dpi, with a decrease of approximately 13% (**Figure 4C**). High levels of viral replication were detected in the lungs (E gene copies approximately 5.0 log10 copies/mg), nasal turbinates (E gene copies approximately 5.7 log10 copies/mg), and brain tissues (E gene copies approximately 7.1 log10 copies/mg) at this time point, with viral replication remaining elevated until the mice succumbed to the infection by 7 dpi (**Figure 4D**). In contrast, mice immunized with M5-RBD adjuvanted with HP007 plus Al(OH)_3_ exhibited no significant changes in body weight (**Figure 4C**), and all survived (**Figure 4B**). The viral titers in various tissues of the vaccinated group were significantly lower than those in the control group (**Figures 4D, E**). At 4 and 7 dpi, E gene copies in the lungs, nasal turbinates, and brains of the vaccinated group were reduced by approximately 2 log^10^ copies/mg, 1–2 log^10^ copies/mg, and 5 log^10^ copies/mg, respectively, compared to the control group. By 14 dpi, viral loads in all tissues approached or fell below the detection limit.

The lung pathology revealed that on 4 dpi with the WT strain (**Figure 4F**), significant thickening of the alveolar walls, a slight reduction in alveolar size, lymphocyte infiltration, and multiple hemorrhagic spots were observed in the lungs of PBS-immunized mice. By 7 dpi, the mice succumbed to the infection, exhibiting pronounced thickening of the alveolar walls, a significant reduction in alveolar cavities, multiple hemorrhagic points in both the alveoli and bronchi, as well as evident interstitial pneumonia. In contrast, the immunized group displayed normal alveolar wall thickness and intact alveolar cavity structure on the 4 dpi, with only minimal hemorrhages present. On 7 dpi, slight thickening of the alveolar walls, a minor reduction in alveolar cavities, and very few instances of bleeding were noted in the lungs of the immunized mice. By 14 dpi, no significant lesions were observed in the lungs of the immunized mice. The lung pathology of all mice was graded according to established criteria (**Figure 4G**). The severity of lung pathological changes was assessed on a scale of 0 to 5, where A score of 0 indicated normal alveolar tissue, A score of 1 represented mild inflammatory cell infiltration without thickening of the alveolar walls, A score of 2 denoted moderate inflammatory cell infiltration with slight thickening of the alveolar walls (1–2 times), and A score of 3 indicated severe inflammatory cell clustering accompanied by thickening of the alveolar walls (2–3 times). Scores of 4 and 5 indicated severe clustering of inflammatory cells, thickening of alveolar walls, bronchiolar obstruction, and consolidation. Consistent with previous findings, lung inflammation levels in the control group mice were higher than those in the vaccine-immunized group. Furthermore, the severity of lung inflammation peaked at the time of mouse death (7 dpi). The degree of tissue pathology in the mice correlated with the trend in viral load. In contrast, mice in the vaccine-immunized group consistently exhibited lower levels of lung inflammation throughout the experiment. On 4 dpi, the PBS group showed bleeding in the nasal turbinate glands and loss of mucosal epithelial cilia. By 7 dpi (**Figure 4H**), the cilia of the epithelium had been shed, and the epithelial cells exhibited signs of bleeding. In the vaccinated group, no significant lesions were observed in the epithelial cells or glands of the nasal turbinate. These results demonstrate that M5-RBD adjuvanted with HP007 plus Al(OH)_3_ can effectively protect mice from SARS-CoV-2 WT virus challenge and significantly reduce the level of viral replication in mouse tissues.

The protective effect of the vaccine against the BA.2 strain challenge was further evaluated. The same immunization and infection processes described previously were employed (**Figure 5A**). Mice were intranasally infected with 5000 PFU of the BA.2 virus. The body weights of The control group began to decrease on 6 dpi, declining by approximately 11% by 7 dpi, followed by continuous weight loss (**Figure 5C**). One mouse in the control group died on 8 dpi, while others succumbed on 9 dpi (**Figure 5B**). In contrast, the body weights of the immunized mice did not exhibit any significant decrease throughout the experiment; instead, they showed a slight upward trend, and all mice in this group survived. The BA.2 strain infection was milder compared to that of the WT strain. the viral titers in the lung tissues of the control group mice displayed a decreasing trend from 4 dpi (with an average of 5.6 log^10^ E gene copies/mg) to 9 dpi (with an average of approximately 4.4 log^10^ E gene copies/mg), while viral loads in the nasal turbinates and brains gradually increased (**Figure 5D**). Specifically, The mean E gene copies in the nasal turbinates rose from approximately 3.8 log^10^ copies/mg to 5.9 log^10^ copies/mg, and the mean E gene copies in the brains increased from approximately 3.1 log^10^ copies/mg to 7.7 log^10^ copies/mg.

The viral titers in various tissues of the immunized group were significantly lower than those observed in the control group (**Figures 5D, E**). At 9 dpi, the E gene copies in the lungs, nasal turbinates, and brains of the vaccinated group were reduced by approximately 1.3 log^10^ copies/mg and 2.8 log^10^ copies/mg, respectively, compared to the control group. By 14 dpi, the viral loads in all tissues approached or fell below the detection limit, with the viral titers in the brain tissues of the immunized group remaining at very low levels throughout the entire infection process. These results indicate that M5-RBD adjuvanted with HP007 plus Al(OH)_3_ can effectively and significantly reduce the replication levels of Omicron BA.2 infection in mouse tissues.

Slight thickening of the alveolar walls and a minor reduction in alveolar spaces was observed in the control group after infection with Omicron BA.2 on 4 dpi (**Figure 5F**), along with lymphocyte infiltration and several hemorrhagic spots. In contrast, the vaccinated group exhibited only a few instances of bleeding, and the basic structure of the alveoli showed no significant pathological changes. By 9 dpi, all mice in the control group had died, and at this time, evident interstitial pneumonia, lymphocyte infiltration, thickened alveolar walls, and a significant reduction in alveolar spaces were noted in their lungs. At this stage, four randomly selected mice from the vaccinated group were euthanized for examination, revealing mild thickening of the alveolar walls and a minimal number of hemorrhagic spots. On 14 dpi, all mice in the vaccinated group were euthanized for sampling, and no obvious pathological changes were observed in their lungs.

At 4, 9, and 14 dpi, the degree of lung tissue damage in the immunized group was significantly reduced compared to the control group (**Figure 5G**). Given that the Omicron strain is more likely to infect the upper respiratory tract (Hui et al., 2022), we further examined the histopathological status of the nasal turbinates in the mice (**Figure 5H**). The results indicated that at 4 dpi, the control group exhibited partial loss of pseudostratified ciliated columnar epithelial cells in the nasal mucosa, whereas the ciliated epithelial cells in the immunized group were tightly arranged and displayed no evident pathological changes. The submucosal gland structure in the control group showed significant cellular damage, which became more pronounced at 9 dpi, leading to an almost complete absence of epithelial cells. In contrast, no significant pathological changes were observed in the nasal turbinates of the immunized mice. Additionally, results from the Omicron infection in immunized BALB/c mice demonstrated that M5-RBD provides superior protection against the Omicron challenge compared to WT-RBD (**Supplementary Figures S3B, C**). Collectively, these data suggest that M5-RBD, when adjuvanted with HP007 and Al(OH)_3_, can effectively protect mice from challenges posed by both the SARS-CoV-2 prototype and the Omicron variant.

## Discussion

SARS-CoV-2 has claimed millions of lives due to its rapid transmission and pathogenicity since its emergence. The emergence of various SARS-CoV-2 variants and cross-species transmission presents significant challenges to the development of vaccines and antivirals (Chen et al., 2020). Unlike pre-Omicron variants (e.g., Alpha, Beta, Gamma, Delta), which carried only 2–3 RBD mutations, Omicron and its sublineages possess over 15 mutations in the RBD. This confers significant immune evasion and represents a fundamental challenge to previous antigen design strategies based on single variants. Introducing viral antigens assists the immune system in recognizing specific epitopes, thereby activating the body’s immune response. However, mRNA vaccines utilizing the Omicron spike/RBD monomer as an immunogen have not demonstrated a significant enhancement in immunogenicity during sequential immunization, whereas the Delta antigen has been shown to elicit effective cross-variant neutralization. Omicron are not more effective than the original vaccine,and natural infection, or specific vaccines developed against it fail to elicit broadly protective immunity against other variants. Collectively, these findings highlight the inherent limitations of vaccine strategies with a narrow focus on a single variant (Waltz, 2022; Li et al., 2022).

To enhance the broad immune response, a common strategy in the development of updated COVID-19 vaccines is the inclusion of spike antigens from immune escape variant strains. Before the emergence of Omicron, the Beta and Gamma variants—both classified as immune escape VOCs, had their S proteins frequently utilized as additional antigens. Moderna’s mRNA-1273.351, which is based on the Beta variant, elicits effective neutralizing antibody responses against the Beta variant in mice when compared to the prototype mRNA-1273. However, it demonstrates reduced efficacy against the Gamma and Epsilon variants (Wu et al., 2021a). Furthermore, Vaccines formulated with S proteins derived from the Beta or Gamma variants appear to exhibit lower neutralization effects against the Delta variant (Ying et al., 2022). Additionally, a vaccine utilizing a Delta-Omicron dimer has been shown to provide broader neutralizing activity compared to the prototype vaccine (Xu et al., 2022). A bivalent vaccine composed of the N-terminal domain (NTD) from the B.1.620 lineage, RBD-S2 from the Gamma variant, and monomeric S protein with other RBD mutations demonstrated broad protective effects in hamster models (Wu et al., 2022).

Early studies on the COVID-19 pandemic indicated that the receptor-binding domain (RBD) exhibits stronger immunogenicity compared to the full-length S, S1, or S2 regions (Yang et al., 2020), research suggests that the RBD is particularly critical for neutralizing antibody responses, with over 90% of neutralizing antibodies targeting this region (Piccoli et al., 2020). RBD dimers fused with Fc tags not only demonstrate enhanced immunogenicity but also offer advantages in protein expression and purification (Liu et al., 2020). Furthermore, the RBD exhibiting the greatest variation among variants is most strongly associated with immune escape compared to other regions. Given these considerations, the RBD-Fc platform was therefore chosen to construct the antigen for vaccine development. On the other hand, based on systematic analysis in **Figure 1C**, most mutations in RBD region show limited impact on ACE2 binding and immune escape to neutralizing antibody, except for few high-impact mutation sites. Therefore, we hypothesize that the precise selection of high-impact mutation sites is key to developing broad-spectrum vaccines.

It should be noted that simply incorporating newly emerging mutation sites or adopting sequences from recent variants does not necessarily enhance the immunogenicity of vaccines against circulating strains (Gagne et al., 2022; Chen et al., 2023). As shown in **Figure 1C**, most RBD mutations—classified as “low-impact mutation sites” and enclosed in black circles—cluster near the origin in both the ACE2 binding and immune escape dimensions. Based on these findings, we propose an antigen design principle that emphasizes “balancing variable and conserved regions.” Guided by this strategy, we constructed the broad-spectrum candidate antigen, M5-RBD, which integrates key mutations including K417T, L452R, T478K, E484K, and N501Y. This design aims to synergistically cover both variable and conserved epitopes, thereby directing the immune system toward a more balanced and broad-spectrum antibody response. The results indicate that, sera from M5-RBD-immunized mice demonstrated not only significantly higher neutralizing titers against D614G, Delta, BA.1, BA.2, and BA.2.75 but also considerable cross-neutralization against emerging variants including BA.5, BF.7, BQ.1.1, XBB, and KP.3, compared to the WT-RBD group (**Figure 2F**). Antigenic cartography demonstrated a reduced antigenic distance between sera from M5-RBD-immunized mice and multiple SARS-CoV-2 variants, indicating that M5-RBD indeed induced a broader neutralizing antibody response.

SARS-CoV-2 exhibits convergent evolution, wherein RBD mutations demonstrate a focused and recurrent pattern across dominant variants. This phenomenon provides a rationale for broad-spectrum vaccine strategies targeting key mutational sites. Notably, although point mutations such as E484K in M5-RBD are replaced in the Delta (which lacks a mutation at site 484) and Omicron BA.1/BA.2 (E484A) lineages, M5-RBD still elicited significantly superior neutralizing activity against these variants compared to M4A-RBD. This indicates that including certain ancestral mutations, even those superseded in later variants, can still induce effective cross-protective immunity. Furthermore, the immunogenicity of M5-RBD was stronger than that of M6-RBD, which carries additional low-impact mutation sites such as N440K or G446S (**Figure 1G**), thereby validating our strategy of screening for high-impact mutation sites.

In addition to optimizing antigens for broad-spectrum vaccine effectiveness, enhancing the functionality of existing adjuvants represents another strategy to improve immune activity. For COVID-19 vaccines, augmenting the role of adjuvants further strengthens the immune response. This strategy encompasses the optimization of adjuvant components, improvements in delivery and release mechanisms, and the enhancement of vaccine stability and durability. Through these approaches, the immune system’s response to the vaccine can be augmented, leading to a more robust broad-spectrum defense. Traditionally used Al(OH)_3_ adjuvants, however, typically activate a weak immune response and do not elicit cellular responses. Consequently, research has focused on optimizing adjuvants through the development of STING pathway agonists, such as CF501, and zinc-aluminum mixed adjuvants modified with bisphosphonate salts, like FH002C (Liu et al., 2022b; Wu et al., 2021b).

In our adjuvant screening, we selected the TLR9-activating CpG adjuvant HP007 to enhance vaccine efficacy. HP007 demonstrated superior immunogenicity over both traditional Al(OH)_3_ and the reference CpG 1018, provoking stronger and more durable humoral and cellular immune responses. A significant T-cell response was maintained for up to four months, highlighting its exceptional ability to induce long-lasting immunity. Research has shown that passive immunity or intramuscular DNA vaccination is insufficient to suppress SARS-CoV-2 infection in the nasal mucosa (Zhou et al., 2021). In the mild BALB/c infection model (Halfmann et al., 2022), the M5-RBD vaccine demonstrated superior efficacy in suppressing viral load in the upper respiratory tract compared to its WT-RBD (**Supplementary Figures S3A–C**), confirming its potential in preventing mild symptomatic infection. Moreover, in the lethal K18-hACE2 mouse model, our subunit vaccine provides effective protection against lethal infections from both SARS-CoV-2 WT and Omicron strains, inhibiting viral infection in the lungs, brains, and nasal turbinates. By converting an otherwise lethal systemic infection into a manageable and localized process, the vaccine effectively prevents severe disease and death. This containment strategy also holds the potential to reduce viral mutation rates, underscoring its significant clinical and broader public health benefit.

This study evaluated the immunogenicity and protective efficacy of M5-RBD in naïve animal models. While the data clearly demonstrate its potential as a vaccine candidate, a key limitation lies in the model’s inability to adequately recapitulate the complex pre-existing immune landscape in humans who have previously been exposed to SARS-CoV-2 through vaccination or infection. This limitation is particularly relevant given our central hypothesis that M5-RBD—through its rationally designed antigenic composition—may effectively recruit pre-existing memory B cells and induce *de novo* immune responses against novel epitopes within the unique immunological context established by inactivated vaccines.

Moreover, the optimal vaccination regimen, including the number of doses and intervals between immunizations, requires further investigation. All currently approved subunit vaccines in China employ a three-dose primary schedule. However, in the context of our study, where M5-RBD is being developed as a booster candidate for individuals with pre-existing immunity, suggesting potential room for optimizing the immunization strategy. Research indicates that even in the presence of immune imprinting by the ancestral strain, individuals can still generate a high proportion of variant-specific neutralizing antibodies upon exposure to Omicron (Yisimayi et al., 2024). We hypothesize that for such pre-primed populations, fewer doses might be sufficient to elicit adequate protective responses. The M5-RBD—whose design integrates both conserved sites and key high-impact mutation sites—is anticipated to exert a dual effect: it is expected to effectively recruit and expand the memory B cell repertoire established by prior immunization (e.g., with inactivated vaccines) against conserved epitopes, while the introduced high-impact mutation sites may further stimulate the production of potent neutralizing antibodies targeting novel epitopes. This could potentially circumvent the strong immune imprinting dominated by the ancestral strain and elicit a broader immune response. Nevertheless, this hypothesis warrants validation through systematic immunization-timing studies. Additionally, the current study did not perform T cell epitope mapping; therefore, it remains unclear whether the observed T cell responses are directed primarily against the engineered mutation sites or against conserved regions within the RBD. Although the strong cross-reactive T cell data support the induction of broad cellular immunity by the vaccine, the precise composition and underlying mechanisms of this response require further elucidation. Furthermore, While this study has confirmed that HP007 elicits superior humoral and cellular immune responses compared to the commercially available CpG 1018, the underlying mechanisms responsible for this enhanced efficacy—such as its precise modulation of cytokine networks and differential activation of specific immune cell subsets—remain important scientific questions warranting further investigation.

Taken together, considerable efforts have been made to develop broad, effective, and accessible vaccines against SARS-CoV-2. Our findings indicate that M5-RBD, which incorporates five selected mutations (K417T, L452R, T478K, E484K, and N501Y), can elicit high titers of broad-spectrum neutralizing antibodies against SARS-CoV-2 and its variants. However, additional mutations may adversely affect the immunogenicity of M5-RBD. Furthermore, a novel CpG adjuvant, HP007, has demonstrated its efficacy in eliciting a durable cellular immune response through the TLR9 pathway. Our results contribute to the ongoing efforts to design broad-spectrum, effective, and durable vaccines for the next generation of COVID-19 immunizations.

## Methods

### Ethics Statement

The animal experiments were conducted by certified personnel at the Center for Animal Experiment of Wuhan University and approved by the Institutional Animal Care and Use Committee (AUPWP20220044). The protocols and procedures for managing infectious SARS-CoV-2 within an Animal Biosafety Level-III Laboratory facility were sanctioned by the Institutional Biosafety Committee (IBC). All samples were inactivated in accordance with IBC-approved standard procedures for the removal of specimens from high containment.

### Comparison of nucleotide sequence similarity of SARS-CoV-2 variants

The nucleotide sequence similarity of SARS-CoV-2 Omicron BA.2 (EPI_ISL_17565946) with other variants was analyzed using SimPlot. the sequences compared to the query sequence are presented on the right. Following the comparison, SimPlot generates a similarity plot employing a Kimuratwo -parameter distance model with a moving window of 200 nucleotides and a shift of 20 nucleotides.

### Correlation coefficient calculation

A total of 397 multiplicative changes in neutralization titers of 10 monoclonal antibodies against full-length or single-point mutant pseudoviruses were extracted from the relevant literature (Cox et al., 2023). The correlation between mutation sites and neutralization efficacy was analyzed using the R programming language. Matrix correlation coefficients were computed using the `cor()` function, and heatmaps were generated with GraphPad software. The monoclonal antibodies analyzed included Amubarvimab (Class 1), Bamlanivimab (Class 2), Bebtelovimab (Class 3), Casirivimab (Class 1), Cilgavimab (Class 2), Etesevimab (Class 1), Imdevimab (Class 3), Regdanvimab (Class 1), Sotrovimab (Class 3), and Tixagevimab (Class 1).

### Plasmid construction

The amino acid sequence of the SARS-CoV-2 Spike protein was retrieved from the NCBI database (protein_id=“YP_009724390.1”). The region spanning amino acids 319–541 corresponds to the SARS-CoV-2 RBD protein sequence utilized in this study. The Fc portion was derived from the constant region of the human IgG1 heavy chain (GenBank: AEV43323.1), referred to as hFc. The sequences of the SARS-CoV-2 RBD and hFc were optimized for codon usage and synthesized. Subsequent plasmids for mutation sites were constructed based on this plasmid.

### Cell culture and transfection

Vero E6 and BHK-21-hACE2 cells were cultured in complete Dulbecco’s modified Eagle’s medium (DMEM; Gibco, USA), supplemented with 10% fetal bovine serum (FBS; ExCell, China).

293F cells were cultured and expanded in SMM 293T-II expression medium (catalog number M293TII). The cells were incubated in a shaker incubator at 120 rpm, 37 °C, and 5% CO_2_. Plasmid transfection was performed using PEI MAX (polysciences, catalog number 24765-100) in a transfection system prepared according to the manufacturer’s instructions, once the cell density reached 2.4 × 10^6 cells/mL with a viability exceeding 98%. Cell viability was monitored daily post-transfection, and 3.5 mL of SMS 293-SUPI cell culture supplement (Yiqiao Shenzhou, catalog number M293-SUPI) was added per 100 mL of culture daily. On 4 dpi, cells were harvested by centrifugation at 4000 rpm and 4 °C for 20 minutes to collect the cell supernatant.

### Protein expression and purification

The His-tagged protein (ACE2-His and WT-RBD-His) was purified using Ni-NTA resin (Qiagen, catalog number L00250), while the Fc -tagged protein was purified using Protein A affinity chromatography media (genscript, China). Size exclusion chromatography was performed using a GE Superdex 200 Increase 10/300 GL column. Protein samples were analyzed by SDS-PAGE, with the buffer for non-boiled samples excluding β-mercaptoethanol. Protein quantification was conducted using the BCA method.

### Adjuvant

Imject Alum adjuvant (Thermo Scientific) consists of an aqueous solution containing 40 mg/mL aluminum hydroxide, 40 mg/mL magnesium hydroxide, and non-active stabilizers. The Al(OH)_3_ adjuvant is prepared in accordance with the Chinese Pharmacopoeia. HP007, PV003, and CpG 1018 were sourced from Jiangsu Taipure Biopharmaceuticals Co., Ltd. The sequence of HP007 is 5’-tcgcgaacgttcgccgcgtacgtacgcgg-3’, while the sequence of PV003 is 5’-tcgcgacgttcgccgacgttcgta-3’. The sequence for CpG 1018 is 5’-tgactgtgaacgttcgagatga-3’. The CpG adjuvant, in freeze-dried powder form, is reconstituted with sterile PBS to achieve a concentration of 1 mg/mL, after which it is aliquoted and stored at -80°C.

### Biolayer interferometry analysis

Biolayer interferometry was conducted using an Octet Red96 instrument equipped with Protein A sensors (ForteBio, Germany). Different mutated RBD-hFc proteins were diluted to 5 μg/mL in Kinetics buffer (KB buffer: 1 × PBS, 0.02% Tween-20). The ACE2-His protein was diluted in KB buffer at concentrations of 500 nM, 200 nM, 100 nM, 50 nM, 25 nM, 12.5 nM, and 6.25 nM. The sensor was combined with RBD-hFc at room temperature for 300s until saturation was achieved, followed by incubation with varying concentrations of ACE2-His protein for 400s. Finally, the sensors were dissociated in KB buffer for 400s and regenerated for subsequent use. A 1:1 binding model was employed, and the binding and dissociation constants between the different mutated RBD and ACE2 were calculated from the binding curves, with R² ≥ 0.98.

### Vaccinations of mice

The specific pathogen-free (SPF) female BALB/c mice (6–8 weeks) were procured from Beijing SibaiFu Corporation and maintained at the Experimental Animal Center of Wuhan University. BALB/c mice (n=6) were intramuscularly immunized with 5 μg of RBD mutant protein, adjuvanted with 20 μg of CpG and 0.26 mg of Al(OH)_3_, administered twice. In the adjuvant comparison experiment, another set of six BALB/c mice received intramuscular immunizations of 5 μg of M5-RBD, either non-adjuvanted or adjuvanted with an equal volume of Imject Alum, 0.26 mg of Al(OH)_3_, 20 μg of HP007, or 20 μg of CpG (PV003, HP007, or CpG 1018) combined with 0.26 mg of Al(OH)_3_, respectively. These Mice were immunized three times, and spleens were harvested post-euthanasia at four months for ICS and BCA analysis. For the dose -dependent experiment, BALB/c mice (n=6) were intramuscularly immunized with M5-RBD (1 μg, 5 μg, or 10 μg), adjuvanted with 20 μg of HP007 and 0.26 mg of Al(OH)_3_, administered twice. Additionally, K18-hACE2 mice were immunized three times with 5 μg of M5-RBD protein, 20 μg of HP007, and 0.26 mg of Al(OH)_3_. Immunizations were conducted with a two-week interval between doses. The PBS group served as the control group. Following each immunization, blood samples were collected from the mice using the retro-orbital sinus method 14 days later. The blood samples were then centrifuged at 4 °C at 4000 rpm for 20 minutes to separate the serum, which was subsequently heat-inactivated at 56 °C for 30 minutes and aliquoted for storage at -80 °C for future testing.

### Pseudovirus neutralization assay

The SARS-CoV-2 pseudovirus neutralization assay was conducted as previously described (Nie et al., 2020). Mouse serum was serially diluted in threefold increments and mixed with an equal volume of SARS-CoV-2 pseudovirus (5000 TCID_50_), followed by incubation at 37 °C for 30 minutes. Subsequently, the mixture was combined with an equal volume of a suspended BHK21-hACE2 stable transfected cell line and cultured overnight at 37 °C. After 16 hours, the cell culture supernatant was discarded, and a luciferase substrate was added to each well. The serum dilution exhibiting 50% neutralization activity was determined by calculating the luciferase enzyme activity, referred to as NT_50_ (50% neutralization titer).

### Authentic SARS-CoV-2 neutralization assay

The SARS-CoV-2 WT strain (IVCAS 6.7512) was obtained from the National Virus Resource at the Wuhan Institute of Virology, Chinese Academy of Sciences. The Delta strain (HB0000054), Omicron BA.1 strain (HB0000428), and Omicron BA.2 strain (HB0000513) were sourced from the Hubei Provincial Center for Disease Control and Prevention. All experiments involving authentic SARS-CoV-2 viruses (S01322040G) were approved by the Biosafety Committee Level 3 (ABSL-3) of Wuhan University. In brief, the serially diluted sera were mixed with 100 TCID_50_ of the virus and incubated at 37 °C for 1 hour. This mixture was then added to Vero E6 cells and incubated at 37 °C for an additional hour. Following the incubation, the mixture was discarded, and 1% methylcellulose was added to the cells, which were then incubated in a CO_2_ incubator for 3 days. After the methylcellulose was removed, crystal violet staining was performed, and the plaque count was determined and analyzed using Graphpad Prism 9 software to calculate the PRNT_50_ (plaque reduction neutralization test titer).

### ELSIA

The ELISA experiment was conducted to measure the IgG levels against the SARS-CoV-2 RBD in mouse sera. In brief, 3 μg/mL of antigen was coated onto the plate and subsequently blocked with 1%BSA. Sera were added at a 1:10^4^ dilution, followed by incubation with an AP-conjugated anti-mouse IgG antibody. Signals were developed using TMB substrate (NCM Biotech, China), and the reaction was halted with 2 N NaOH. The absorbance was measured at 405 nm.

### Flow cytometry

Two months after the last immunization, mice were humanely euthanized and splenocytes were harvested and subsequently incubated with 5 μg/mL of RBD protein for 24 hours. Brefeldin A (BFA) was added at a final concentration of 5 ng/mL to treat the cells, five hours before the end of stimulation. The cells were stained with the following surface antibodies: PerCP-Cy5.5™ hamster anti-mouse CD3e (BD, 551163), FITC anti-mouse CD4 (BioLegend, 100406), and APC anti-mouse CD8a (BioLegend, 100712). Subsequently, the cells were fixed and permeabilized, followed by staining with PE anti-mouse IFN-γ (BioLegend, 505808). Data were collected using a DxFlex flow cytometer (Beckman, USA) and analyzed with FlowJo 10.8.1 software.

### Cytometric Bead Array

Immunized BALB/c mice were also euthanized two months after the last immunization. Splenocytes were incubated with 5 μg/mL of RBD protein for 48 hours. Cytokines were detected using the Mouse Th1/Th2 Cytokine Kit (BD, USA) according to the manufacturer’s instructions. In brief, supernatants were collected and incubated with diluted capture beads. After the addition of PE detection reagent, the beads were washed and resuspended for further processing. Data were collected on the DxFlex flow cytometer (Beckman, USA) and analyzed using FCAP assay software.

### Challenge of SARS-CoV-2 in mice

Heterozygous B6/JGpt-H11_em1Cin (K18-ACE2)_/Gpt mice (K18-hACE2 mice, #T037657, under a C57BL/6J background) were purchased from Gempharmatech Co., Ltd. (Nanjing, China). The experiments involving infection with SARS-CoV-2 were conducted in the Animal Biosafety Level 3 (ABSL-3) laboratory at Wuhan University. K18-hACE2 mice (n=12) were intramuscularly immunized with 5 μg of M5-RBD adjuvanted with HP007 plus Al(OH)_3_, administered twice a week for a total of three doses. PBS was used as a control (n=8). After immunization, mice were transferred to the ABSL-3 laboratory for the challenge experiment. They were anesthetized in a closed induction chamber using 4% isoflurane delivered in medical-grade oxygen via a precision vaporizer, and maintained under anesthesia throughout the intranasal inoculation procedure. The mice then inoculated 50 µL of either 250 PFU of SARS-CoV-2 WT or 5000 PFU of the Omicron BA.2 strain. Body weights were monitored daily. Four mice from each group were randomly selected for euthanasia at 4 dpi for further analysis. Additionally, four mice from the immunized group were euthanized on the same day that all mice in the control group succumbed to the infection. The remaining immunized mice were observed until 14 dpi, and tissues were collected following euthanasia. inactivated samples were then transferred to a Biosafety Level 2 (P2) laboratory for further analyses.Viral RNA from tissues was extracted using the RNA Extraction Kit (Vazyme, RM201). Reverse transcription was performed with the Reverse Transcriptase Kit (zomanbio, ZR102). Each reaction system included 15 μL of RNA and 5 μL of 4×RT Mix. The reaction mixture was incubated at 45 °C for 15 minutes and subsequently stored at -20 °C. The SARS-CoV-2 N and E genes were quantified by RT-qPCR using specific primers and probes. Hematoxylin and eosin (H&E) staining was conducted by Wuhan Pinuofei Biological Technology company.

### Statistics

Asterisks were employed to indicate the statistical significance of differences between groups: *P < 0.005; **P < 0.001; ***P < 0.0001; ****P < 0.0001.

## Data Availability

The raw data supporting the conclusions of this article will be made available by the authors, without undue reservation.

## Supplementary material

Supplementary Figure 1 Composition and distribution of mutation sites in the S protein of the variant strain.

Supplementary Figure 2 RBD protein properties assay.

Supplementary Figure 3 Protection effect of the vaccine against SARS-CoV-2 Omicron BA.2 infection in BALB/c mice.
