# Supplementary material for: A broad-spectrum SARS-CoV-2 RBD vaccine with selected high-impact mutations and novel adjuvant induces durable T cell response and broad protection in mice
Source: Front Cell Infect Microbiol. 2026 Jan 30;15:1690554. doi: 10.3389/fcimb.2025.1690554 (PMC12901505; doi:10.3389/fcimb.2025.1690554)
Supplement: Supplementary Figure 1 — Composition and distribution of mutation sites in the S protein of the variant strain. [file DataSheet1.docx]

**Supplementary Figures**

Fig S1. Composition and distribution of mutation sites in the S protein of the variant strain.

Fig S2. RBD protein properties assay.

Fig S3. Protection effect of the vaccine against SARS-CoV-2 Omicron BA.2 infection in BALB/c mice.


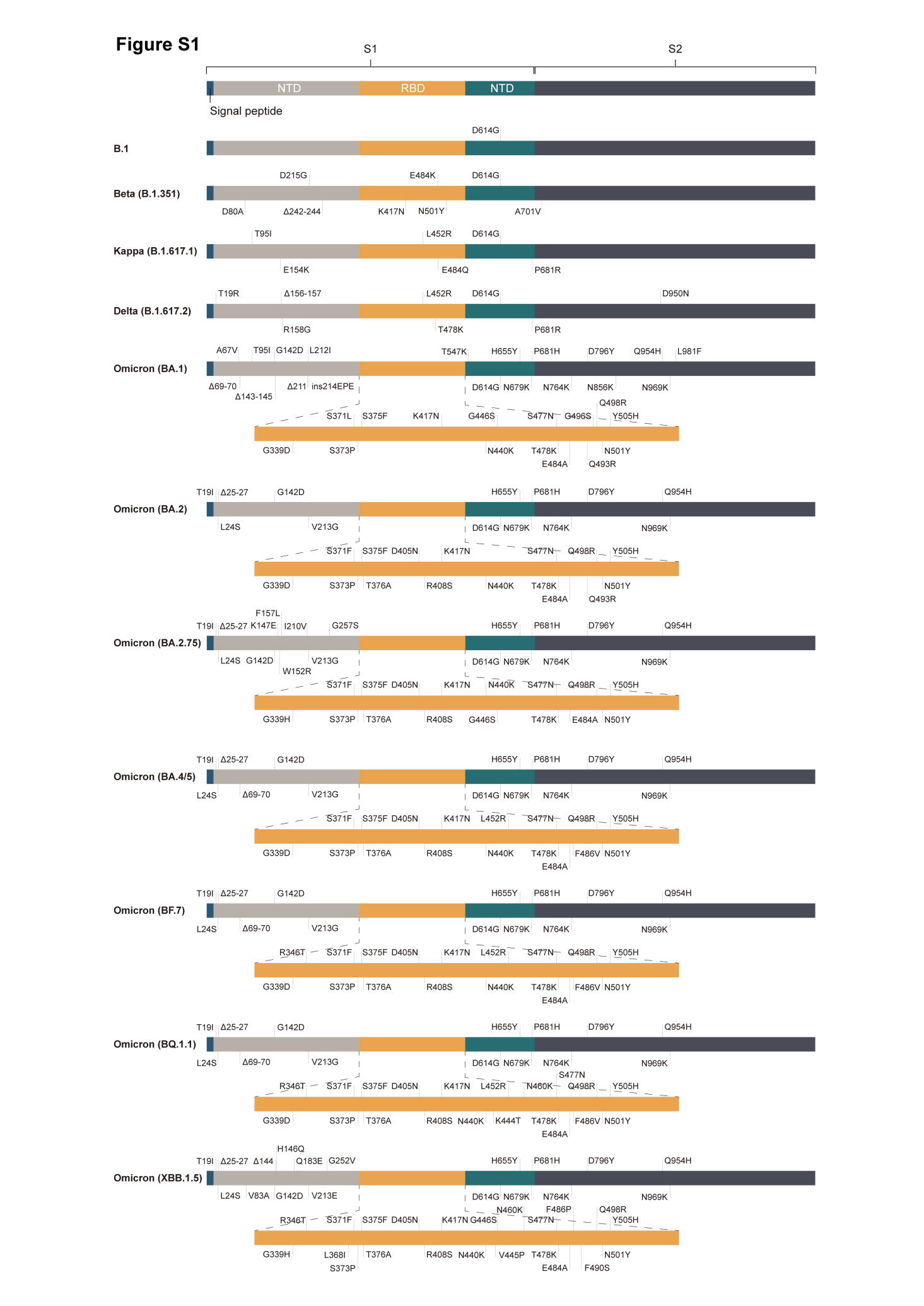


**Fig S1. Composition and distribution of mutation sites in the S protein of the variant strain.**


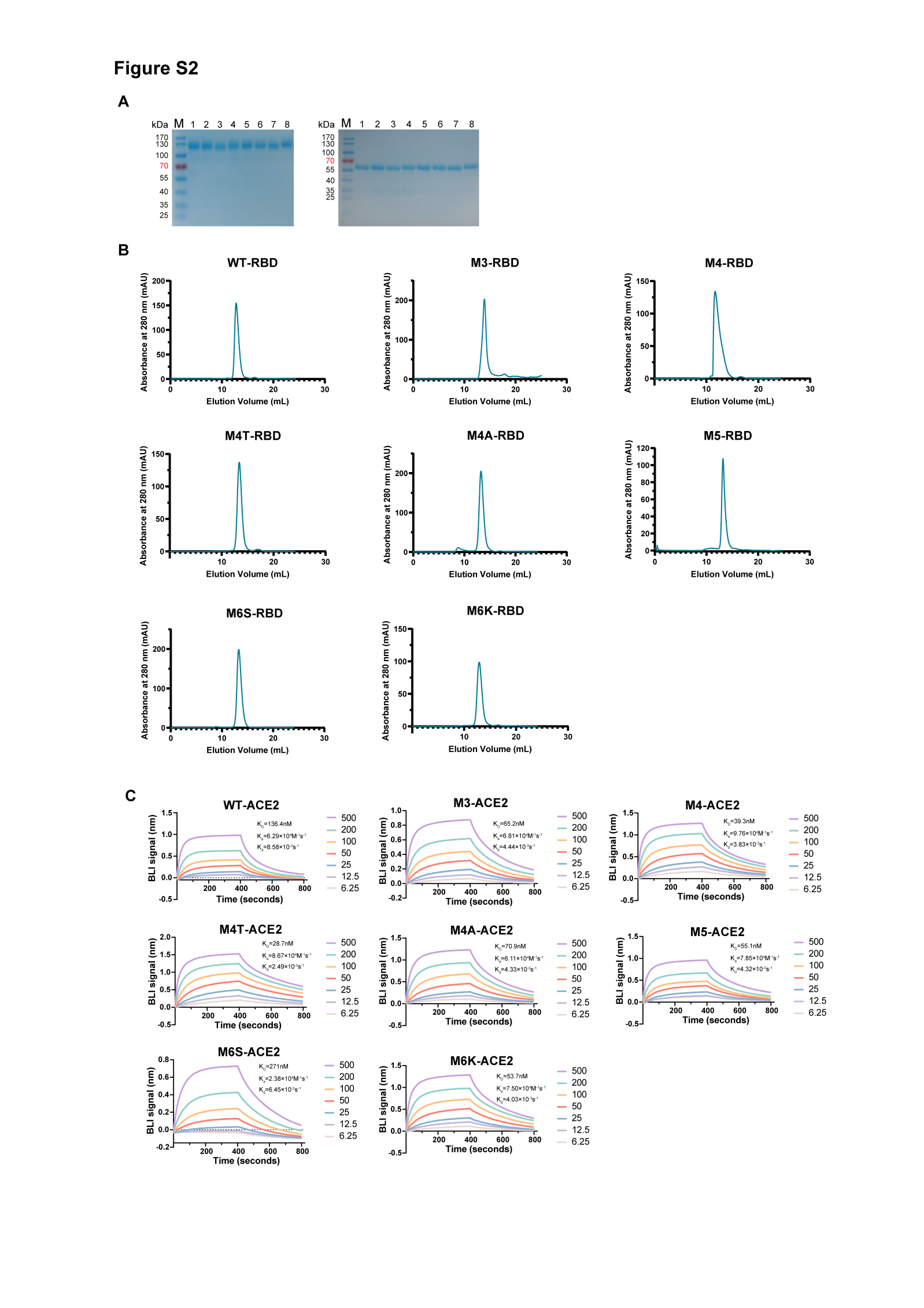


**Fig S2. RBD protein properties assay.**

Eight kinds of RBD featuring various combinations of mutation sites, including M3, M4, and M4T RBD, were expressed in 293F suspension cell lines. The Cell supernatants were purified using affinity chromatography and size exclusion chromatography. A. Expression of 8 kinds of RBD antigen verified with polyacrylamide gel electrophoresis (left: reduced SDS-PAGE; right: non-reduced SDS-PAGE). B. Purification of 8 kinds of RBD antigen after size exclusion chromatography. C. The affinity between the recombinant mutant protein RBD-Fc and the hACE2 receptor was assessed by biolayer interferometry (BLI) .


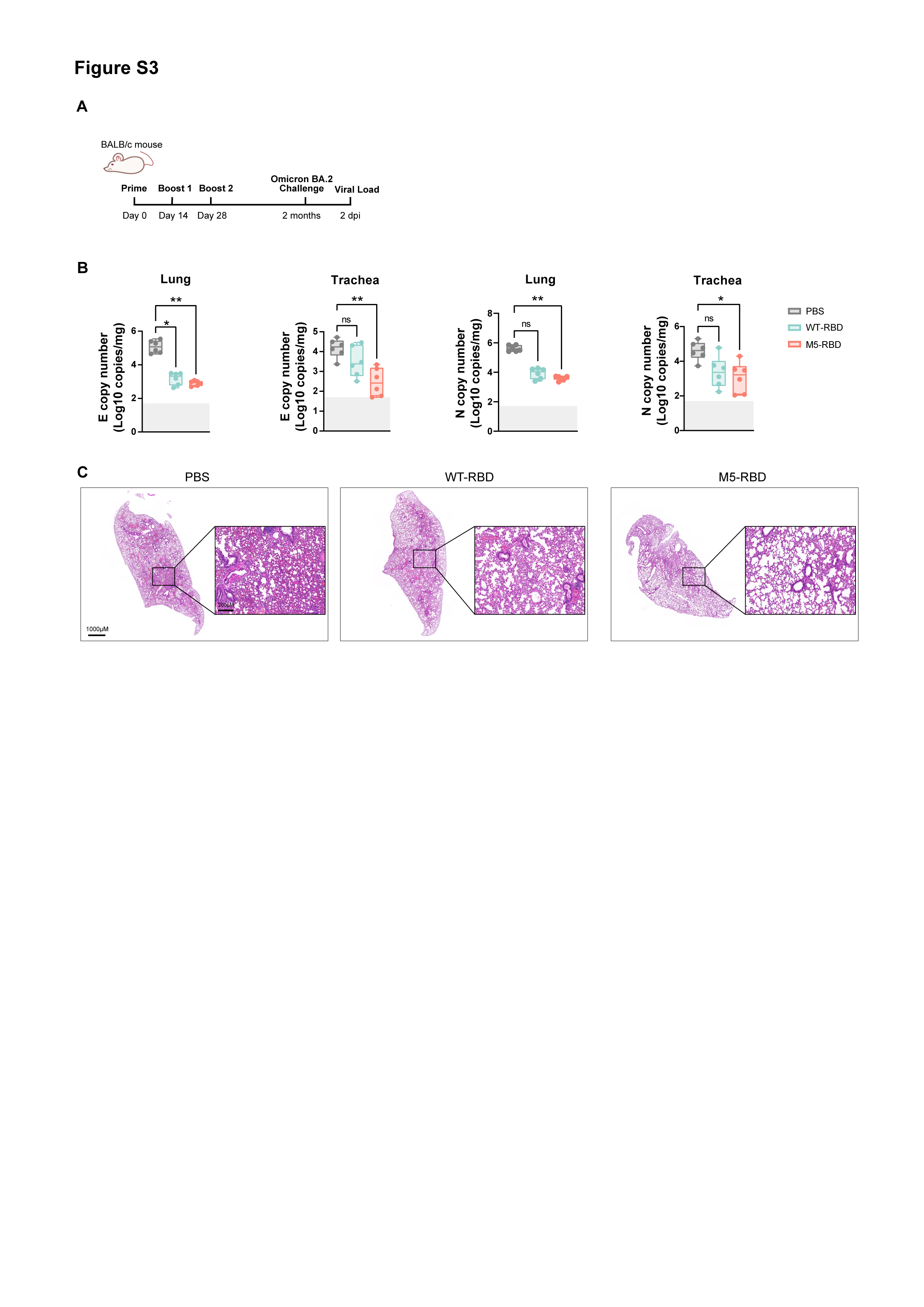


**Fig S3. Protection effect of the vaccine against SARS-CoV-2 Omicron BA.2 infection in BALB/c mice.** BALB/c mice, aged 6-8 weeks, were immunized three times before being subjected to intranasal infection with 1.6 × 10^5^ PFU of the SARS-CoV-2 Omicron BA.2 strain. A. Timeline of the mouse experiment. B. Copy numbers of the SARS-CoV-2 E and N gene in the lung and trachea tissues of the mice after the viral challenge. C. The evaluation of lung pathology through scoring of H&E stained lung sections. Each experimental group consisted of six mice (n=6). Asterisks indicate statistical significance, with "***" representing P < 0.001.
